# RADseq population genomics confirms divergence across closely related species in blue coral (*Heliopora coerulea*)

**DOI:** 10.1186/s12862-019-1522-0

**Published:** 2019-10-15

**Authors:** Akira Iguchi, Yuki Yoshioka, Zac H. Forsman, Ingrid S.S. Knapp, Robert J. Toonen, Yuki Hongo, Satoshi Nagai, Nina Yasuda

**Affiliations:** 1 Department of Bioresources Engineering, National Institute of Technology, Okinawa College, 905 Henoko, Nago-City, Okinawa 905-2192 Japan; 2 Present address: Geological Survey of Japan, National Institute of Advanced Industrial Science and Technology, AIST Tsukuba Central 7, 1-1-1 Higashi, Tsukuba, Ibaraki 305-8567 Japan; 3 Hawai’i Institute of Marine Biology, Kaneohe, HI USA; 4 Research Center for Aquatic Genomics, National Research Institute of Fisheries Science, 2-12-4 Fukuura, Kanazawa-ku, Yokohama, Kanagawa 236-8648 Japan; 5 Department of Marine Biology and Environmental Sciences, Faculty of Agriculture, University of Miyazaki, Gakuen- kibanadai-nishi-1-1, Miyazaki, 889-2192 Japan

**Keywords:** Coral, Transcriptome, Ecological divergence, Speciation

## Abstract

**Background:**

*Heliopora coerulea*, the blue coral, is the octocoral characterized by its blue skeleton. Recently, two *Heliopora* species were delimited by DNA markers: HC-A and HC-B. To clarify the genomic divergence of these *Heliopora* species (HC-A and HC-B) from sympatric and allopatric populations in Okinawa, Japan, we used a high throughput reduced representation genomic DNA sequencing approach (ezRAD).

**Results:**

We found 6742 biallelic SNPs shared among all target populations, which successfully distinguished the HC-A and HC-B species in both the sympatric and allopatric populations, with no evidence of hybridization between the two. In addition, we detected 410 fixed SNPs linking functional gene differences, including heat resilience and reproductive timing, between HC-A and HC-B.

**Conclusions:**

We confirmed clear genomic divergence between *Heliopora* species and found possible genes related to stress-responses and reproduction, which may shed light on the speciation process and ecological divergence of coral species.

## Background

Reef-building corals are morphologically and ecologically diverse and per unit area the reefs they form support more species than any other marine ecosystems [1]. Coral reefs are also among the most threatened ecosystems from direct and indirect anthropogenic pressures [2, 3]. *Heliopora coerulea*, the blue coral, is the only octocoral to form a massive structure like scleractinians and due to its characteristic blue skeleton it is also harvested for the aquarium, jewelry and curio trade. The blue coral is also only found in the shallow waters of the Indo-Pacific, making it particularly susceptible to long-term climate change and local anthropogenic impacts [4, 5].

Species delineation is one of the most fundamental issues when assessing conservation and biodiversity strategies, but morphological species identification of reef-building corals is often problematic due to their high plasticity and limited number of species-specific features [6], which is also applicable to octocorals [7]. Molecular techniques provide approaches to better inform these phylogenetic relationships in corals (e.g., [8–10]). However, these molecular studies often suffer from a paucity of relevant markers to elucidate detailed evolutionary processes in corals (reviewed by [11]). In particular, mitochondrial DNA (mtDNA), is often used to infer inter- and intra-specific differences in many animal species (reviewed in [12, 13]); however, it has slow mutation rates in corals [14, 15]. Species delineation is particularly difficult with closely related corals because of interspecific hybridization, recent speciation, shared ancestral polymorphisms, and/or extremely high intra-specific morphological variation [16–22].

Nuclear markers have sometimes been more useful and have played an important role in understanding the phylogenetic and geographic relationships of corals (e.g., [7, 18, 23–26]). However, inadequate taxonomy, discord between nuclear and mitochondrial results, hybridization or incomplete lineage sorting, cryptic species, and difficulty in distinguishing population level genetic structure from species level genetic structure all complicate efforts to resolve species boundaries in corals (e.g., [21, 27–29], reviewed by [30]). The development of high-throughput reduced representation genomic DNA sequencing provides the opportunity to easily examine hundreds to thousands of nuclear markers as short loci or single nucleotide polymorphisms (SNPs). RADseq has also been applied to octocorals [22, 31]. There are now several RADseq protocols making it easier and cost-effective to perform SNP analyses on non-model organisms including corals (e.g., [32–35]).

In this study, we applied the ezRAD [32, 36] approach to examine two closely related groups of *Heliopora* (Pallas 1766) corals on the southern reef of Okinawa, Japan, which reproduce at different times [37] and are often found in different habitats [38]. These two *Heliopora* species were recently delimited by microsatellite markers [25] and the ITS2 region [26]: HC-A and HC-B. This study aims to expand on this previous work to search for biallelic fixed SNPs in functional genes to further clarify the relationship between these octocoral *Heliopora* species.

## Methods

### Sampling, DNA extraction and library preparation

**Fig. 1:**
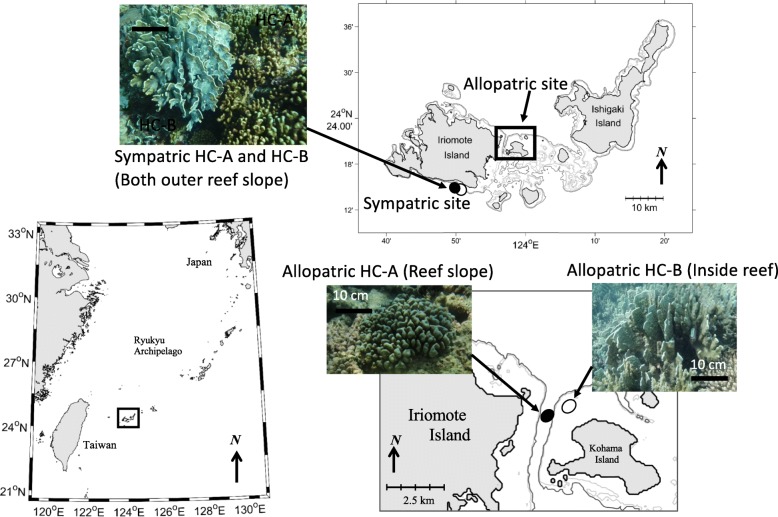
Sampling locations of *Heliopora* species used in this study

We selected four populations of two *Heliopora* spp. (each population was collected from two allopatric sites and one sympatric site; Fig. 1). The coral fragments (1–2 cm) were collected either by snorkeling or on SCUBA (depth: 0.8–7.8 m) as described in [25] under a permission from Okinawa Prefecture (26–10). Genomic DNA was extracted immediately after sampling of coral fragments with a Qiagen DNeasy Blood and Tissue Kit. Each of the four populations had twelve individuals (Table 1), which were quantified with the Accuclear Ultra High Sensitivity dsDNA kit before pooling equimolarly. DNA samples from 12 individuals in each site were pooled and used for the following analyses. The four libraries were prepared following the ezRAD protocol [36] using Illumina TruSeq library preparation kit, and following bioanalyzer and qPCR quality control steps were run as paired-end (2 × 300 bp) reads on the Illumina Miseq sequencer.

**Table 1:** Summary of used samples in this study

	Allopatric HC-A	Allopatric HC-B	Sympatric HC-A	Sympatric HC-B
No. of individuals	12	12	12	12
No. of reads (paired-end, forward)	4,552,218	3,585,919	4,599,520	3,793,508
No. of reads (paired-end, reverse)	5,022,763	3,974,309	5,084,202	4,193,414

### Bioinformatics analysis

The FASTQ files, with an average of 8.7 million 300 bp reads per paired-end population, were filtered with the FASTX-Toolkit (http://hannonlab.cshl.edu/fastx_toolkit/) to discard reads with poor quality bases (Q < 20) and less than 25 bp in length. To increase the efficiency of mapping against transcriptome data, we prepared fasta files consisting of 50 bp size sequences from filtered fastq files above. In order to call SNPs in putative protein coding regions from the coral host, we aligned the fasta files to the transcriptome data of coral host *Heliopora coerulea* [39] with bowtie2 by using the default setting [40]. This process excludes contaminated sequences from other micro-organisms for the following analyses. With the subsequent SAM files, we called SNPs with Stacks (programs: pstacks (default setting), cstacks (−b 1 -p 4 -n 3), sstacks (−b 1 -p 4), and genotypes (−b 1) [41];). Short read data is available from the accession No. DRA008338 (DNA Data Bank of Japan (DDBJ)).

Based on catalog files made by Stacks, we prepared input files including only biallelic SNPs loci among 4 populations using parsing scripts by R ([42]; Additional file 1) for the following analyses. Based on the biallelic SNPs data, hierarchical clustering of the number of loci, in which two populations share the same allelic compositions and heat map visualization, were performed using heatmap.2 in the gplots ver. 3.0.1 package in R [43]. Venn diagram was drawn using VennDiagram ver. 1.6.17 package in R. We also performed a maximum likelihoood (ML) analysis with RAxML ver. 8.2.7 [44] using NEXUS file including concatenated biallelic SNPs data. For the analysis, we used the GTR-GAMMA model and 1000 bootstrap replicates to estimate the clade confidences. Using short sequences obtained by Stacks including SNPs that were alternately fixed between types (HC-A and HC-B) found by parsing scripts by R (Additional file 1), we performed BLASTN analysis (e-value cut-off: 1e^− 5^) against transcriptome sequences of *H. coerulea* published in a previous study [39] and obtained annotation information for each SNP. We performed all data processing and analyses using the supercomputer of the National Institute of Genetics (Mishima, Shizuoka, Japan).

## Results and discussion

**Fig. 2:**
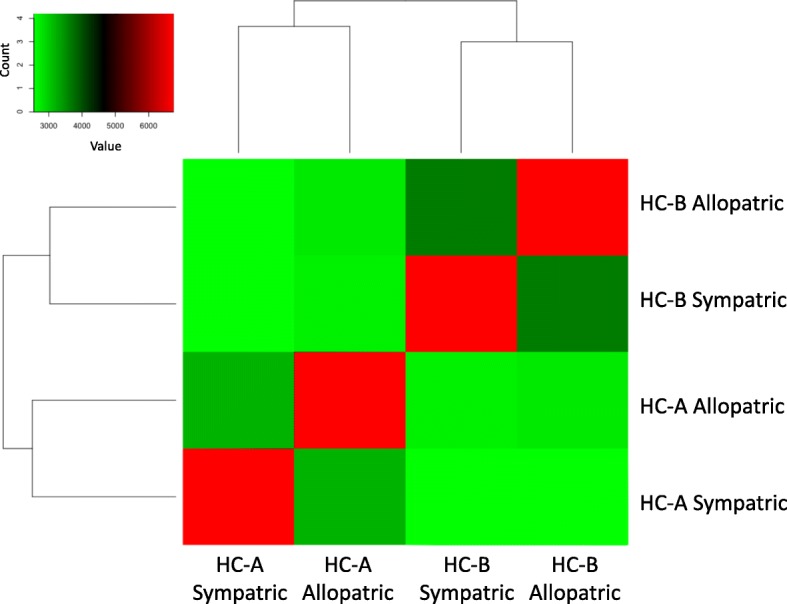
Heat map of the number of loci in which two populations share the same allele compositions

**Fig. 3:**
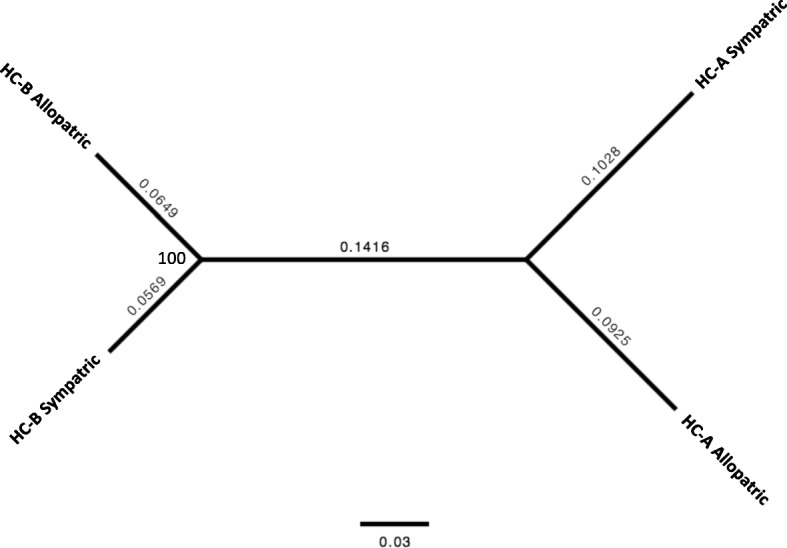
Dendrogram of the four populations of *Heliopora* libraries. The node values show ML-bootstrap percentage and the values above the branches are genetic distances (substitutions/site)

The ezRAD libraries yielded on average 8.7 million 300 bp reads per population (Table 1). After excluding contaminated sequences by using transcriptome data of *H. coerulea*, we succeeded in detecting 6742 variable biallelic SNPs shared among all 4 of the pooled populations. The number of loci at which pools of individuals shared the same nucleotide was higher within species (3199 and 3631 in HC-A and HC-B, respectively) than between species (2556–2750) regardless of locations (Fig. 2, Additional file 2). Based on the SNP polymorphisms, the dendrogram indicates that the HC-A and HC-B species remain clearly distinguished regardless of whether they were collected from sympatric or allopatric populations (Fig. 3). This distinction supports the previous population genetic analyses using microsatellite and ITS2 markers [26], indicating that, even in sympatric environments, there is either striking selection or no hybridization between HC-A and HC-B as suggested in previous studies [25, 26]. But considering that we used pooled RAD-seq samples, individual based analysis would be necessary in the future [45].

In addition to the population genetic distinction, we also detected 410 fixed different SNPs in sequences obtained by Stacks between the two species (HC-A and HC-B). A BLAST search revealed matches to 314 annotated genes from the coral host (almost all e-values <1e^− 10^; Additional file 3). The BLAST results included genes related to stress responses such as thioredoxin [46], ubiquitin-protein ligase [47, 48], and cryptochrome-1 as a candidate gene (Table 2) although these SNPs were located at synonymous positions. Bay et al. [47] reported that SNP mutation in cryptochrome-1 was potentially linked to heat resistance in populations of *Acropora hyacinthus*. In the well-developed fringing reef, HC-A is more commonly found on the colder outer reef slope compared to HC-B, which resides in warmer shallower waters. Indeed, distribution of HC-A is also further north than that of HC-B [49]. In addition, after a mass bleaching event in 2017, more HC-B survived than HC-A in Sekisei Lagoon (H. Kurihara and N. Yasuda unpublished data). Thus, the fixed nucleotide differences between HC-A and HC-B provide hypotheses for the underlying mechanisms of differential stress tolerances observed between these species. This stress tolerance should be further examined, because the resilience of these two *Heliopora* species to long-term climate change will likely differ and be an important component of future conservation and management strategies.

**Table 2:** Selected candidate genes for ecological divergence of *Heliopora* species

Contig ID	Accession number	Annotate description	Origine speices	Accession number	Identity (%)	E-value	Bit score
c101459_g1_i1	IABP01020958	PREDICTED: dopamine receptor 2-like	*Acropora digitifera*	XP_015754865.1	43.64	2.00E-21	99.4
c114522_g1_i1	IABP01022095	PREDICTED: LOW QUALITY PROTEIN: E3 ubiquitin-protein ligase RNF103-like	*Acropora digitifera*	XP_015753316.1	41.68	2.00E-164	498
c31599_g1_i1	IABP01003528	E3 ubiquitin-protein ligase HECTD3	*Exaiptasia pallida*	KXJ16705.1	56.16	0	959
c41722_g1_i2	IABP01005816	Thioredoxin domain-containing protein 11	*Exaiptasia pallida*	KXJ26582.1	32.17	2.00E-113	385
c48657_g1_i1	IABP01009691	PREDICTED: E3 ubiquitin-protein ligase UBR5-like isoform X8	*Parasteatoda tepidariorum*	XP_015929469.1	42.2	0	908
c52917_g1_i2	IABP01016181	PREDICTED: E3 ubiquitin-protein ligase RNF213	*Callorhinchus milii*	XP_007886854.1	35.62	0	1979
c61162_g1_i1	IABP01017641	Cryptochrome-1	*Exaiptasia pallida*	KXJ26519.1	45.79	0	932

Interestingly, dopamine receptor 2-like gene was also found among the fixed SNPs gene list, which has been linked to the season an animal breeds [50]. The timing of reproduction is different between HC-A and HC-B [37, 50]. For example, in both the Philippines [51] and Japan (Taninaka et al. under review), HC-A broods their larvae about 1 month earlier than HC-B even in sympatric sites, indicating that reproductive timing of *Heliopora* spp. appears to be genetically controlled rather than environmentally-dictated. It is reported that dopamine is related to the spawning timing of *Acropora tenuis* [52]. In addition, it is suggested that cryptochrome-1 is involved in reproductive timing of acroporid coral [53]. Thus, it is possible that these fixed genetic differences in the dopamine receptor and cryptochrome-1 might contribute to the difference of reproductive patterns in *Heliopora* spp. and highlight the need for additional research.

## Conclusions

We detected clear divergence between *Heliopora* species based on SNPs obtained from the ezRAD approach utilizing coral host transcriptome data. These data indicate that even among sympatric populations, HC-A and HC-B are reciprocally non-interbreeding, and therefore warrant formal recognition as valid taxonomic species. We also highlight candidate genes which may explain ecological differences between HC-A and HC-B, especially those found in the sympatric populations, which provide mechanistic hypotheses for the divergence of these groups and suggest likely differences in stress response and resilience to future climate conditions. More detailed descriptions of ecological characteristics such as reproduction and stress tolerances between HC-A and HC-B, guided by hypotheses based on fixed SNP differences discovered in this study, will contribute to a deeper understanding of the mechanistic and genetic basis of the ecological divergence of blue corals and the speciation process.

## Supplementary information

**Additional file 1.** R script for analyzing SNPs data.

**Additional file 2.** Venn diagram showing the numbers of loci in which populations share the same allele compositions.

**Additional file 3: Table S1**. Candidate genes for ecological divergence of *Heliopora* species.

## Data Availability

All data of this study are included in this article and its supplementary information.

## Abbreviations

DNA: DeoxyriboNucleic Acid
ITS: Internal Transcribed Spacer
PCR: Polymerase Chain Reaction
RADseq: Restriction Site Associated DNA Sequence
SNP: Single Nucleotide Polymorphism
