# Supplementary material for: RADseq population genomics confirms divergence across closely related species in blue coral (Heliopora coerulea)
Source: BMC Evol Biol. 2019 Oct 15;19:187. doi: 10.1186/s12862-019-1522-0 (PMC6794731; doi:10.1186/s12862-019-1522-0)
Supplement: Supplementary file 1 — Additional file 1. R script for analyzing SNPs data. [file 12862_2019_1522_MOESM1_ESM.pdf]

```
### R script to extract biallelic SNPs data from tsv file made by
Stacks ("concensus" of tsv file should be removed prior to the
following treatment) ###

d <- read.delim("<tsv file>", sep="	",
stringsAsFactors=FALSE, header=T, na.strings="")

# Select only Catalog ID and SNPs data
d4 <- d[,c(1, 4 ,5 ,6 ,7)]

# Extract SNP loci including only 2 alleles
n <- which(apply(d4,1,function(x) min(nchar(x))==1),TRUE)

d4b <- d4[n,]

d4e <- t(d4b)

CatalogID1 <- d4e[1,]

d4e <- d4e[-1,]

y <- length(d4e[1,])
t <- length(d4e[,1])

# Convert alleles into numerical values
M <- NULL
for(j in 1:y){
D1 <- c(subset(d4e,select=j))
D2 <- strsplit(D1, "/")
for(i in 1:t){
if(length(D2[[i]])==1){
D2[[i]] <- c(D2[[i]],D2[[i]])
}
if(length(D2[[i]])==2){
D2[[i]]
}
m <- unlist(D2)
o <- c(1:t)
m1 <- m[2*o-1]
m2 <- m[2*o]
m3 <- cbind(m1,m2)
}
M <- cbind(M, m3)
}

# Add Catalog ID
CatalogID2 <- rep(CatalogID1, each=2)
CatalogID3 <- paste(CatalogID2,
rep(c("1","2"),length(CatalogID1)),sep="_")
colnames(M) <- CatalogID3

Md <- as.data.frame(M)

N <- vector()
```

```
for(k in 1:y){
Me11 <- M[,2*k-1]
Me12 <- M[,2*k]
l <- length(unique(c(Me11,Me12)))
N <- c(N,l)
}
summary(N)
length(which(N==2))
hist(N)

# Convert only biallelic locus into numerical values
p <- NULL
for(k in 1:y){
Me11 <- as.character(Md[[2*k-1]])
Me12 <- as.character(Md[[2*k]])
u <- unique(c(Me11,Me12))
O <- cbind(Me11, Me12)
colnames(O) <- c(colnames(Md[2*k-1]), colnames(Md[2*k]))
if(length(u)!=1){
if(length(u)==2){
O <- sub(u[1], "1", O)
O <- sub(u[2], "2", O)
}
p <- cbind(p, O)
}
}

# Extract column including only numerical values
Q <- t(p)
R <- as.data.frame(Q)

# Extract rows including only "1" and "s"
Re <- subset(R, V1 == "1" | V1 == "2")

# Confirm the number of locus
(length(Re[,1]))/2

### R script to extract fixed locus between HC-A (assuming rows 1
and 3) and HC-B (assuming rows 2 and 4) ###

Ree <- t(Re)

Rd <- as.data.frame(Ree)
for(i in 1:((length(Ree[1,])-2)*0.5)){
if((
(Ree[1,(2*i+1)]==Ree[1,(2*i+2)])&
(Ree[1,(2*i+1)]==Ree[3,(2*i+1)])&
(Ree[1,(2*i+1)]==Ree[3,(2*i+2)])&
(Ree[1,(2*i+2)]==Ree[3,(2*i+1)])&
(Ree[1,(2*i+2)]==Ree[3,(2*i+2)])&
(Ree[3,(2*i+1)]==Ree[3,(2*i+2)]))
&
((Ree[2,(2*i+1)]==Ree[2,(2*i+2)])&
(Ree[2,(2*i+1)]==Ree[4,(2*i+1)])&
```

```
(Ree[2,(2*i+1)]==Ree[4,(2*i+2)])&
(Ree[2,(2*i+2)]==Ree[4,(2*i+1)])&
(Ree[2,(2*i+2)]==Ree[4,(2*i+2)])&
(Ree[4,(2*i+1)]==Ree[4,(2*i+2)]))
&
(Ree[1,(2*i+1)]!=Ree[2,(2*i+1)])
)
{print(dimnames(Rd[2*i+1])[[2]])}
}
```
