# Supplementary figures and images for: RADseq population genomics confirms divergence across closely related species in blue coral (Heliopora coerulea)
Source: BMC Evol Biol. 2019 Oct 15;19:187. doi: 10.1186/s12862-019-1522-0 (PMC6794731; doi:10.1186/s12862-019-1522-0)

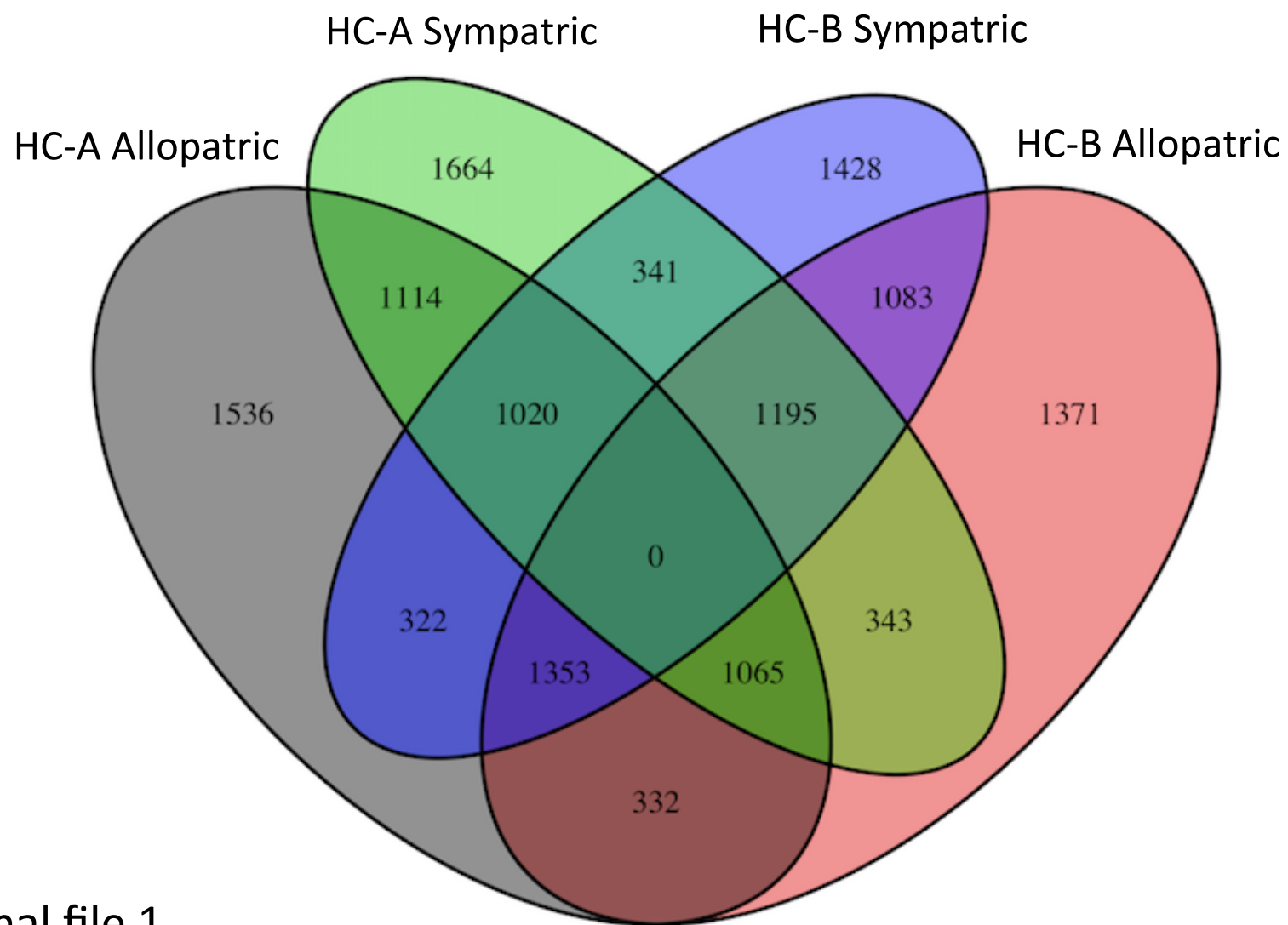

Additional file 1

Supplement: Supplementary file 2 — Additional file 2. Venn diagram showing the numbers of loci in which populations share the same allele compositions. [file 12862_2019_1522_MOESM2_ESM.pdf]
